# The Role of Systemic Filtrating Organs in Aging and Their Potential in Rejuvenation Strategies

**DOI:** 10.3390/ijms23084338

**Published:** 2022-04-14

**Authors:** Amal Kassab, Nasser Rizk, Satya Prakash

**Affiliations:** 1 Biomedical Technology and Cell Therapy Research Laboratory, Department of Biomedical Engineering, Faculty of Medicine, McGill University, 3775 University Street, Montreal, QC H3A 2BA, Canada; amal.kassab@mail.mcgill.ca; 2 Department of Biomedical Sciences, College of Health Sciences-QU-Health, Qatar University, Doha 2713, Qatar; nassirzk@qu.edu.qa

**Keywords:** aging, rejuvenation, kidney, filtration organ, plasma proteomics, heterochronic parabiosis, metabolic pathways, urine proteomics, metal clearance, aging biomarkers

## Abstract

Advances in aging studies brought about by heterochronic parabiosis suggest that aging might be a reversable process that is affected by changes in the systemic milieu of organs and cells. Given the broadness of such a systemic approach, research to date has mainly questioned the involvement of “shared organs” versus “circulating factors”. However, in the absence of a clear understanding of the chronological development of aging and a unified platform to evaluate the successes claimed by specific rejuvenation methods, current literature on this topic remains scattered. Herein, aging is assessed from an engineering standpoint to isolate possible aging potentiators via a juxtaposition between biological and mechanical systems. Such a simplification provides a general framework for future research in the field and examines the involvement of various factors in aging. Based on this simplified overview, the kidney as a filtration organ is clearly implicated, for the first time, with the aging phenomenon, necessitating a re-evaluation of current rejuvenation studies to untangle the extent of its involvement and its possible role as a potentiator in aging. Based on these findings, the review concludes with potential translatable and long-term therapeutics for aging while offering a critical view of rejuvenation methods proposed to date.

## 1. Introduction

Aging literature has shown remarkable advances over the past two decades and has produced significant milestones that might allow for the first time to view aging as a disease that is not only treatable but also reversible [1,2,3]. However, these claims remain to date under dispute, as sustainable aging reversal methods are yet to be identified, and in the absence of universally accepted aging biomarkers that successfully isolate healthy aging from disease phenotypes associated with it, its classification as a disease is premature. Thus, although aging is still not officially classified as a disease, it is still a prominent risk factor for a multitude of chronic ones. Admittedly, when it comes to geroscience, it is hard to sum up, findings in the field using clear and comparable metrics, largely since it is very diverse, spanning lifestyle factors [4,5,6,7], dietary recommendations [8,9,10,11,12], pharmaceutical interventions [13,14], and young blood plasma transfusions [15], in addition to cell reprogramming agents [16,17,18]. Additionally, most current literature on the topic ignores the global process of aging, specifically its primary driver and chronological progression, instead of focusing on specific manifestations of it. However, the biggest challenge in assessing current findings on aging is the lack of a common quantifier of biological age. To date, most rejuvenation studies measure the reversibility of some molecular and genetic markers of certain aging-associated chronic diseases or parameters, while the use of systemic cellular and molecular quantifiers of biological aging remains confined to a limited number of studies. This might be due to the fact that measures of biological aging are themselves diverse and continuously advancing, as some rely on assessing circulating C-reactive protein (CRP), p16^INK4a^, or telomere attrition, the latter two being also common biomarkers of cellular senescence [19,20,21,22,23]. The most promising biological aging quantifier, however, seems to be DNA methylation, which was presented through several aging “clocks,” such as Horvath’s or Hannum’s, that helped introduce lifespan predicting ones, such as PhenoAge and GrimAge [24,25]. Despite challenges in adopting such methodologies into broader clinical use, as a definite measure of biological age today, the promise of an accurate and precise biological age quantifier is undoubtedly closer than ever, which would be the ultimate judge on the success of any advocated rejuvenation method. Nevertheless, until a unified platform and parameters in geroscience are set, assessing the value of these largely scattered findings remains taxing, especially via well-established scientific methods, such as meta-analysis. More importantly, the effort to bridge these findings to attain a complementary understanding of the systemic phenomenon of aging is becoming increasingly demanding due to the exponential increase of publications in the field.

There are several excellent reviews that detail the recent advances made in all rejuvenation strategies, including quantifiers of biological aging, or age metrics, some of which have already been referenced in the introduction. Therefore, this review attempts to shine a light on a possible systematic aging factor that has been overlooked thus far. Moreover, by critically reviewing the latest findings on aging and rejuvenation strategies, we propose new avenues to address aging and to understand its chronological progression. In the following sections, we start with a juxtaposition between the aging of biological and mechanical fluidic entities. This over-simplification primarily aims to provide an overview of the major systemic causes of aging, in order to break down this phenomenon into main categories that can be targeted and studied. This primary analysis clearly indicates that the filtration system presents a clear gap in aging studies to date. Next, hallmarks of biological aging are discussed, where several milestone publications were able to identify clear aging phenotypes on molecular, cellular, and organismal levels. Nevertheless, no clear understanding of aging progression over time, specifically primary causes and downstream targets, is available to date. Following this analysis and based on the gap in addressing the role of systemic filtration on aging, the role of the kidney specifically on various known aging targets is reviewed, namely the role of the kidney in handling metabolic by-products, metal, circulating aging factors, and plasma clearance. Next, a critical review of present rejuvenation strategies is provided to assess their long-term significance and practicality. Finally, we conclude by exploring possible future rejuvenation avenues that need to be developed to address the complex topic of healthy aging. 

## 2. An Engineering Perspective on Biological Aging

The degradation of physical entities, whether biological or non-biological, over time can be regarded as one of the fundamental natural laws, specifically the second law of thermodynamics. Nevertheless, biological systems are exceptionally resilient due to their built-in maintenance and renewal capabilities that defy non-biological ones in their complexity and efficiency. These systems, however, are far from perfect and eventually exhibit systemic malfunctions that lead to various errors/diseases and finally degrade, leading to death.

**Figure 1:**
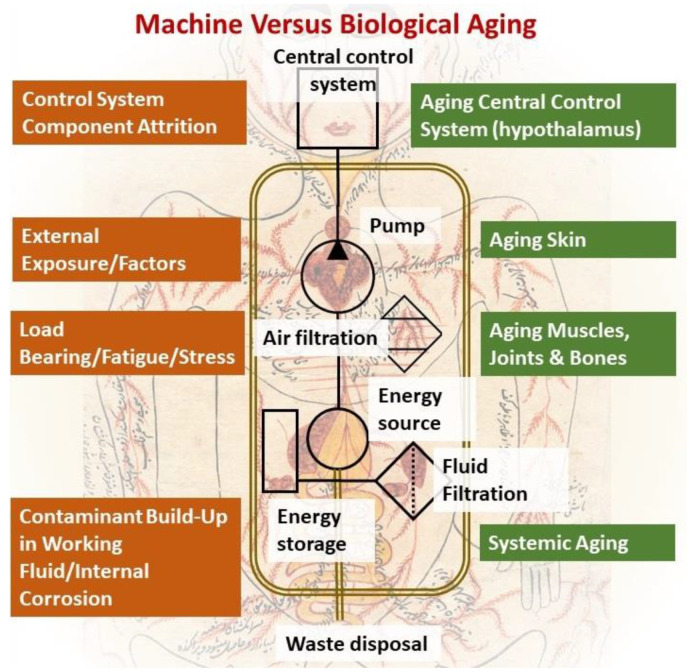
Causes of aging: Juxtaposition between mechanical fluidic and biological systems.

However, compared to the vastly diverse and complicated biological systems, mechanical fluidic ones are much simpler and rudimentary, which in a topic as complicated and diverse as aging brings about a much-needed simplicity that can potentially provide a unified platform to assess and measure this process in a more organized and structured manner. The aging of mechanical devices can be ascribed to a set of simple parameters, such as fatigue and stress-induced on moving and load-bearing parts. Additionally, in fluidic systems, the aging of mechanical parts is due to either external environmental exposure or internal corrosion due to a buildup of contaminants within the circulating fluid. These contaminants tend to accumulate over time on internal surfaces, compromising component integrity and eroding their protective and insulating layers due to undesirable chemical reactions, as shown in Figure 1. Therefore, the most important measures to ensure a long service life of any fluidic system rely on using good quality materials, protecting against external environmental factors, and maintaining a robust and changeable filtration system. Notably, the more complex, and sensitive the fluidic device’s components are, the more sophisticated filtration systems become to ensure a secure and safe operation of all internal components.

Based on this analogy, external body parts are obviously susceptible to accelerated aging due to environmental stress, such as UV light, heat, cold, and possible reactionary agents [26,27]. On the other hand, moving and load-bearing parts, such as joints, the heart, muscles, and bones are subject to wear and tear [28,29]. This leaves behind the systemic cause of aging, where fluid integrity that is largely controlled by the filtration system is in question. In biological systems, where organ functions are not as clear cut as in their mechanical counterparts, the single most comparable organ in charge of fluid filtration is the kidney, which seems to have the highest burden in protecting the entire system from contamination build-up. Yet, interestingly, the kidney as an organ has never been considered for its possible role in physiological aging thus far, despite the clear correlation between the functional decline of the kidney and physiological aging in humans. 

Reduced kidney function generally starts in the late thirties, which is concomitant with the onset of aging, and continues to degrade over the years; nevertheless, it is not considered an issue from a medical standpoint until the loss of function reaches more than 50% [30,31]. Furthermore, while the effect of the loss of kidney function beyond the critical threshold is well documented, there is no account for the effect of reduced kidney function on the body in general and whether this gradual damage plays a role in the initiation or acceleration of the biological clock. Kidney disease, or the reduction in its functionality beyond 50%, is associated with inflammation, oxidative stress, sympathetic–vagal imbalance, and circadian rhythm, in addition to tissue atrophy, increased protein catabolism, and the activation of the stress resistance response [32], all of which are directly related to aging. Additionally, of the limited chronic diseases known to cause premature biological aging, such as heart failure, obstructive pulmonary disease, rheumatoid arthritis, and HIV, kidney disease is one of the most common [33,34,35,36,37]. Aging hallmarks of these diseases include muscle atrophy, vascular disease, and general frailty [32,35,36], which might be attributed to the sedentary lifestyle that often accompanies chronic diseases. Nevertheless, these diseases also share cellular markers of aging and senescence, namely telomere attrition and p16^INK4a^ accumulation [38]. 

Of course, correlation alone does not establish causation, especially when considering the complex nature of the aging process and its interpersonal variations. Therefore, a closer re-assessment of aging hallmarks and successful “rejuvenation” methods are warranted to elucidate the possible extent of the kidney’s involvement in aging.

## 3. Hallmarks of Aging: The Complexity of Cause and Effect

**Figure 2:**
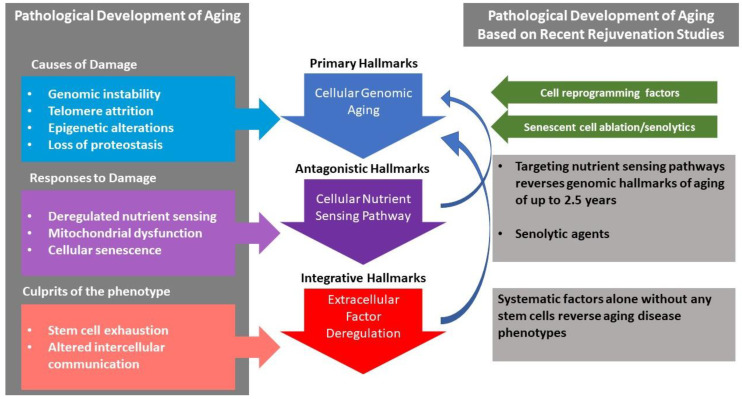
The pathology of aging and the causative relationship between its hallmarks. An illustration of the differences between theoretical assumptions regarding the hierarchical order of the aging process [39] and the various experimental avenues that show the complex relationship between the various hallmarks of aging. These include targeting nutrient sensing pathways [6,7,44,45,46,47], circulating factors and young plasma [48,49,50,51], senescent cell ablation [52,53,54,55,56] and cell programming factors [57,58].

In general, aging hallmarks are consistent on physiological, organ, cellular, and molecular levels. Several review articles have covered aging hallmarks comprehensively [20,39,40], therefore, they will not be detailed herein, rather, the association between these hallmarks will be viewed. Early on, in 2013, Lopez-Otin et al. proposed dividing the hallmarks of aging into primary hallmarks identified as genomic instability, telomere attrition, epigenetic alterations, and impaired proteostasis, as the main initiators of the aging process. This is followed by another set of aging hallmarks as a response, which manifest in deregulated nutrient sensing, mitochondrial dysfunction, and cellular senescence that eventually lead to integrative aging hallmarks such as stem cell exhaustion and altered intercellular communication [39], as shown in Figure 2. This work by Lopez-Otin et al. presents one of the earliest and most comprehensive reviews on aging, with a rare insight into the possible hierarchical and chronological sequence of the aging process that has been the cornerstone of many studies since. The timing of the article also reflects a transitional period in aging research between the two opposing views that regarded this process either as unidirectional or reversible. This might be behind the interpretation that the aging process is fundamentally cellular in nature, and that somatic cell exhaustion is behind the global aging phenomenon that is exacerbated by stem cell exhaustion at its later stages. While this view holds merit in some of its details, it has been challenged by rejuvenation research that has been flourishing at the same time, particularly those pertaining to heterochronic parabiosis, where extracellular factors alone that are devoid of any stem cells were shown to have rejuvenating effects capable of reversing the hallmarks of cellular and molecular aging [41,42,43]. In other words, extracellular factors might be implicated in renewing or maintaining endogenous cellular repair mechanisms. Thus, experimental evidence that rejuvenation studies provide, may allow for an overview of the aging process’s hierarchy to discern the root cause of the phenomenon, and ultimately address the source of the disease itself. 

## 4. Circulating Factors of Aging and the Kidney 

The most obvious drawback of targeting certain “aging” pathways is indeed the complexity and subtle role of the variation of these pathways in different cell types and tissues, which hinders such methods from becoming a widely adaptable and long-term solution for aging. To be clear, while none of the targeted approaches seem to provide a solution to the accumulation of contaminants within the bloodstream, in the absence of a cure for a malfunctioning filtration system, reducing the build-up of metabolic waste with the adoption of a restricted calorie intake, for example, can understandably decelerate the aging process, in addition to the various other factors that add up to achieve the effects observed using such approaches. In this regard, the kidney primarily functions as a purifier of metabolic by-products and a maintainer of plasma mineral homeostasis; additionally, it also filters out small to medium size proteins, all of which have been shown to affect biological aging in one aspect or another.

### 4.1. Metabolic by-Product Clearance and Kidney-Associated Protein Metabolism

It is known that the estimated glomerular filtration rate (eGFR), which is a measure of serum creatinine levels, reduces gradually with age, even among healthy people [44]. In fact, according to one study in an Italian population, the prevalence of reported kidney disease is merely 3.3% in the group with eGFR < 60 mL/min /1.73 m^2^ [45]. Reduced eGFR is shown to be significantly associated with uric acid, urea, and free ammonia accumulation, particularly citrulline, glycine, and phenylalanine [46]. Based on recent findings, elevated levels of serum uric acid alone, or hyperuricemia, has been linked with gout [47], bone and tissue damage [48], heart disease [49], high blood pressure [50], fatty liver [51], and type 2 diabetes [52], all of which are associated with aging. Therefore, it is worth considering that aging can be defined as an ailment that grows underneath clinically defined disease thresholds, much like contaminant levels within the blood stream. Nevertheless, there are additional metabolic factors involved in reduced kidney function. In a recent study by Chen et al. (2020), comparative blood and urine metabolomics analysis was conducted that identified 32 significantly altered compounds between healthy young and elderly groups [59]. The most noteworthy of which were decreased serum levels of albumin lysyl and essential amino acids, and increased levels of N-acetyl glycoproteins and lipids. On the other hand, urine samples of the elderly had elevated levels of trimethylamine N-oxide, scyllo-inositol, citrate, and ascorbic acid, with decreased levels of amino acids and acetate, among others. Taking into consideration that this is the first age-associated study that compares variations between serum and urine, it provides a rare insight into possible causative factors involved in metabolic changes associated with aging. Nevertheless, the limited number of participants (33 in each group) emphasizes the importance of conducting more comprehensive research in this area based on larger groups of participants in different populations. While there is no doubt that progressive loss of kidney function leads to changes in protein/amino acid exertion, changes in kidney metabolism itself might be implicated in the deregulation of vasoactive compounds and hormones leading to health complications in the elderly [60]. Such changes can be used as possible aging markers, based on urine samples alone, according to a recent study by Teruya et al. (2020) that identified 55 possible metabolic markers, most of which are highly correlated with creatinine [61]. Additionally, age-related changes in kidney-specific transcriptomics can provide additional insight on its role in systemic aging [62].

### 4.2. Urine Metal Concentration and Aging

Although protein metabolism and its by-product clearance have well-founded effects on aging and age associate diseases in general, the role of mineral homeostasis and the effect of reduced kidney function is less evident. Nevertheless, based on several studies, urine metal clearance increases with age systematically, allowing it to be used as a predictor of biological aging, particularly iron, zinc, and manganese clearance [62,63]. The importance of increased metal clearance can be demonstrated by the role that some metals play in various organic functions, such as hormone production, blood oxygenation, and enzyme formation.

Iron is one of the most important non-organic elements for a biological system. Iron deficiency has been associated with decreased cognition and functional ability in the elderly, which might be a downstream effect of reduced blood oxygenation [64] that is by far the most important role of iron. The prevalence of anemia increases with age, starting with 17% up to 45% in the nursing home elderly population [65]. On the other hand, the importance of zinc stems from it being an essential component in various biological processes, such as genomic repair [66], growth and reproduction [67], protection against inflammation [68], immunity [69], wound healing, and enzyme synthesis [70], in addition to its important role in maintaining central nervous system function [71]. Additionally, the serum zinc/copper ratio is associated with bone mineral density, which implicates the detrimental effect of reduced serum zinc levels on skeletal health [45]. Finally, when it comes to manganese, unlike iron and zinc, its biological role is limited to trace amounts for various biological functions including enzyme synthesis [46]. Additionally, since it is available in most diets, its excess expression in urine is associated with toxic exposure, which leads to several neurodegenerative diseases [47]. In fact, according to Nwanaji-Enwerem et al. (2020), manganese can be considered an important phenoage as a 1 ng/mL increase in its concentration in urine is associated with a 9.93 year increase in biological age defined by DNA methylation clocks [44]. 

Most studies on urine metal concentration are presently within the realm of toxicology, however, given the importance of certain metals in biological function, the effect of aging on metal clearance and the specific role those various organs play in the process of their circulation need to be clarified. Presently, based on the limited number of studies on this topic, metal clearance seems to increase with age, which might enforce and/or propagate age-related disease progression, without much evidence supporting its role in the onset of aging.

### 4.3. Circulating Factors and Plasma Protein Clearance by the Kidney

There have been several extensive studies that undertook the examination of plasma protein variations with age as a result of the rejuvenating effects observed with heterochronic parabiosis. Nevertheless, studies on urine profile variation in connection with age are less common outside of the aim of identifying disease phenotypes as a diagnostic tool. 

When it comes to plasma protein disparities with aging, one interesting study presented by Lehallier et al. (2019) was able to identify 1379 proteins that changed with age [72]. More importantly, the stratification of changes in these protein concentrations over time provides a rare insight into their association with the aging process itself. The authors remarked that while some groups of proteins seem to be somewhat stable during various life-stages and only start to increase exponentially at the onset of older age (> 60 years), other “clusters” accelerate exponentially around mid-age (late 30–40 years), and some tend to increase/decrease linearly with age, as shown in Table 1. This remarkable analysis identifies for the first-time proteins and their associated pathways implicated in the aging process itself versus those that are a product of an aging system and a manifestation of its malfunction. Based on this examination, it is obvious that clusters 3, 4, 7, 8, and to a lesser degree 5, represent proteins involved in the aging process. Specifically, clusters 3, 4, and 7 present proteins that increase/accumulate in the plasma over time, while clusters 5 and 8 decrease with age. Table 1 details the main pathways implicated in these clusters, outlined in the supplementary data provided by Lehallier et al. [72]. Apart from this important and rare global view of plasma protein changes, individually, the most significantly age-associated proteins that are gender-independent can be reduced to, sclerostin (SOST), growth differentiation factor 15 (GDF15), ADP ribosylation factor interacting protein 2 (ARFIP2), motilin (MLN), pleiotrophin (PTN), and scavenger receptor class f member 2 (SCARF2) that increase with age, while the expression of immunoglobulin superfamily DCC subclass member 4 (IGDCC4) and proto-oncogene tyrosine-protein kinase receptor (RET) decrease over time.

Some of these individual proteins come as a confirmation for the results published by an earlier study in 2018 by Tanaka et al., which presented another plasma proteomic analysis of aging in healthy humans [73]. This investigation identified 197 proteins in connection with aging, 20 of which are negatively associated. The top 10 most significantly affected proteins are GDF15, PTN, a disintegrin and metalloproteinase with thrombospondin motif 5 (ADAMTS5), follitropin (CGA-FSHB), SOST, chordin-like protein 1 (CHRDL1), natriuretic peptides b (NPPB), EGF-containing fibulin-like extracellular matrix protein 1 (EFEMP1), macrophage metalloelastase (MMP12), and cathepsin (CTSV). Additionally, these previously identified circulating plasma factors include the detrimental decrease with age of: growth differentiation factor 11 (GDF11) [74], oxytocin (OXT) [75], tissue inhibitor of metalloproteinase 2 (TIMP2) [76], granulocyte-macrophage colony-stimulating factor (CSF2) [76], growth hormone-releasing hormone (GHRH) [77], apelin (APLN) [78], cadherin 13 (CDH13) [79], extracellular nicotinamide phosphoribosyl transferase (eNAMPT) [80], and thrombospondin-4 (THBS4) [81], in addition to osteocalcin (OCN) [82]. Circulating factors that instead increase with age include small cytokines (CCL2, CCL11 and CCL19) [83], haptoglobin (HP) [83], and β-2 microglobulin (B2M) [84]. In this regard, taking into consideration that old blood seems to have a more damaging effect on organs and tissues than young blood, more emphasis can be dedicated to those that accumulate over time. 

When it comes to the general decline in kidney function and its effect on protein clearance, to date, there is only one comparative proteomic analysis of age-associated changes in urine, which included urine samples of 52 healthy men aging 19–54 years [85]. The study identified 259 urinary proteins and analyzed their tissue origin and function, out of which 23 increased and one decreased with age. Although the list of major differentially expressed proteins in urine does not correspond with major ones detected in plasma, however, there seems to be a strong correlation between the molecular weight and the amount of protein clearance with age, which can be associated with increased “leakage”, as the kidney loses its protein retaining ability [85]. Therefore, an investigation into plasma protein molecular weight can elucidate this filtration organ’s role in their signature over time. Table 2 lists major plasma proteins affected by age and their molecular weight. Interestingly, apart from SCARF2, all proteins that increase with age within the bloodstream seem to have a small (< 40 kDa) to medium (40–100 kDa) molecular weight. On the other hand, while some small and medium molecular weight proteins seem to decrease with age, large molecular weight plasma proteins (> 100 kDa) that decrease with age include IGDCC4, RET, and THBS4. Additionally, the tissue origin of urine proteins that significantly correlate with age can be traced back to the gonads and bones, then the liver, the pancreas, the kidneys, the spleen, and the soft tissue, followed by the intestines and the larynx, in descending order [85]. 

On the other hand, investigating the possible association of a number of identified plasma proteins with kidney function may lead to insights into the possible involvement of renal function in a mechanistic way. However, such inferences can only be confirmed via direct investigations to verify their causative association with an aging phenotype. For example, higher serum levels of SOST, one of the proteins that increases with age, is associated with reduced GFR, body mass index, male gender, and diabetes, in addition to heritability [86]. Additionally, a chronic heart failure marker, namely GDF15, correlates with parameters of kidney function [87,88]. Interestingly, β-2 microglobulin, one of the most well-established plasma factors of aging that is associated with inflammation [52], cardiovascular events [53], impaired cognitive function [84], and even tumorigenesis [54], has been shown to directly correlate with GFR [55]. This cellular metabolic product that is normally filtered out directly via the glomerulus, accumulates over time in the system as a function of reduced kidney function, to a point that it can arguably be used as a novel GFR marker [55]. Another circulating factor that has been heavily studied as an aging marker in relation with neural degeneration is eotaxin-1 (CCL-11) [56], which belongs to a chemokine family and is linked to inflammation and allergic reactions [57]. Nevertheless, although increased levels of cytokines and chemokines in plasma are largely regarded as systemic senescence parameters of aging, interestingly, according to one in vivo study, cytokine production increases due to induced bilateral nephrectomy, concomitant with decreased cytokine clearance [58]. Additionally, according to several studies, levels of cytokines and chemokines in plasma can be used as markers of kidney disease [89].

In conclusion, the intertwined role of kidney function into the various hallmarks of aging is clear; however, its causative association with aging requires rigorous investigation in order to elucidate the exact role of this filtration organ on the aging phenotype.

## 5. Rejuvenation Strategies: Systemic versus Targeted

From an engineering perspective, systemic factors, as those associated with the working fluid and its integrity, seem to hold precedence over other rejuvenation methods, based on the aging analysis presented earlier. Such a view is backed up by several publications on the rejuvenating effects of young plasma on age-related diseases in animal models [89,103,104,105]. Nevertheless, despite their success, these results are yet to be translated into clinical use, as presently their efficacy hasn’t been demonstrated in the limited number of clinical trials conducted thus far [106,107]. Alternatively, assessments of the main drivers of these positive effects led to the discovery of “circulating rejuvenation factors” in blood plasma [108,109,110]. Interestingly, investigations from Berkeley University led by Dr. Conboy’s group demonstrated that without the benefits of “shared organs” in heterochronic parabiosis animal models, the effect of young plasma in old animals is different, although still present. In addition, an accelerated aging effect due to old blood is more pronounced than the rejuvenating effects of young blood [111]. More importantly, a recent study by the same group demonstrated that replacing half of the plasma of old mice with a mixture of saline and albumin is sufficient to produce rejuvenating effects that meet, or even exceed, those observed in heterochronic parabiosis [112]. The importance of such findings cannot be underestimated, as it outlines the effect of contaminant buildup in the bloodstream on aging and disease manifestations for the first time. Additionally, it might provide a more translatable clinical solution to plasma transfer, which is riddled with issues of compatibility, possible contamination, and limited resources. What remains to be clarified though is the effect such methods have on the biological clock itself, and how frequent blood “purification” procedures would be required to maintain a youthful/healthy status.

Clinical experience from dialysis patients, as well as the few clinical trials that have tested the concept of plasma exchange with specific components, show that improvements come in a seesaw fashion that is often exhausting and inflicts organismal fatigue that may be detrimental to the general system homeostasis [15,112,113,114]. This brings back into question the efficacy of highly aggressive intermittent interventions versus a more constant and stable long-term solution and redirects the discussion towards the possible effect of “shared organs” in heterochronic parabiosis models. In particular, it is worth addressing the role of filtration organs such as the kidney, as they are directly implicated in clearing toxic buildup from the blood stream. As such, would a kidney-specific rejuvenation scheme be the ultimate answer to a translatable medical solution for aging? It is clear by now that there is an association between kidney function and some identified plasma circulating factors that are implicated in aging. Nevertheless, it is also worth evaluating the increasing body of targeted rejuvenating agents that have shown promising results, with some managing to turn the aging clock of DNA methylation by 2.5 years [115]; namely, the nutrient-sensing pathway, cell senescence and a senescence-associated secretory phenotype (SASP), in addition to epigenetic interventions.

### 5.1. Targeting Nutrient-Sensing Pathways

Aging and two of the most prominent chronic diseases mostly associated with it, diabetes and obesity, share the deregulation of nutrient sensing pathways and sporadic low-grade inflammation. A deregulated metabolism has been shown to be a function of the activation of high nutrient-sensing pathways, such as insulin and insulin-like growth factor 1 (IGF-1), that in turn target the forkhead box O transcription factor (FOXO) and mechanistic target of rapamycin (mTOR) complexes. This is accompanied by the downregulation of low nutrient-sensing ones, such as AMP-activated protein kinase (AMPK) and Sirtuins [116]. The effect of these pathways on cellular aging has been solidified by countless studies that stretch back to the earliest rejuvenation investigations based on calorie restriction (CR) [8]. In this regard, it has been demonstrated that blocking one of the pathways or gene targets of IGF-1 or mTOR can be linked with increased energy expenditure, enhanced activity of brown adipose tissue, and improved mitochondrial oxidative metabolism [117]. In fact, these effects have been observed previously in dietary restriction with regards to longevity across all species, which is believed to shift cellular metabolism towards maintenance instead of reproduction and growth [118]. Additionally, mTOR signaling is a key regulator of cell cycle arrest and autophagy [119], which is essential in suppressing the accumulation of senescent cells, making it an interesting target for both senolytic and nutrient-sensing rejuvenation schemes. Moreover, while insulin and IGF-1 pathways are naturally decreased in normal, as well as in accelerated aging, their forced inhibition seems to correlate with longevity and rejuvenation [120]. 

Other nutrient-sensing pathways have also been investigated heavily in relation to aging, including those that decrease with age, such as Sirtuins that rely on NAD+, a critical coenzyme in metabolism [121]. Pharmacological enhancement of NAD+ supplements has shown beneficial effects on a range of metabolic diseases, nevertheless, singling out this pathway has also been linked with increased SASP and tumorigenesis associated with the accumulation of senescent cells and the inflammatory response, all of which contribute to aging [122].

To be sure, nutrient-sensing pathways have been the most well-established avenue thus far when it comes to healthy aging, with several documented clinical trials and data to back up the advantages and trade-offs associated with it. The most promising pharmaceutical interventions targeting these pathways to date are Rapamycin [123], Metformin [124], and Resveratrol [125], to name only a few. Nevertheless, it is important to keep in mind that targeted therapies, especially pharmaceutical interventions that are used continuously, often come with shortcomings that would eventually limit their benefit. This is not surprising, given the complex and intertwined nature of nutrient signaling pathways with those of the cell cycle, apoptosis, and autophagy. Rapamycin for example, which is the most restricted drug within this category, is a known immunosuppressant and used as a part of the chronic regiment of organ transplant patients, which provides an obvious set of drawbacks for its continuous use. 

On the other hand, while studies on the possible involvement of Metformin in dementia and memory loss are contradictory [126,127], its role in diminishing the absorption of vitamin B12 resulting in possible nerve damage or anemia, is well documented [128]. Additionally, Metformin is implicated in lactic acidosis and kidney damage, which might be aggravated by reduced kidney function [129,130]. While these risks are not considered a major concern for diabetes patients since their condition is already a risk factor for nerve damage and inflammation, the use of this drug for longevity purposes might be controversial without a proper long-term assessment of its effects. Conversely, Resveratrol is a natural phenol found in many plants, making it a widely available food supplement that has been shown to improve health, including renal and cognitive functions [131] rather than demonstrating tangible effects on the lifespan [125]. More importantly, effective doses used in aging literature far surpass the daily doses available in the market. Thus, as promising as these interventions may be, even the most benign of which, such as caloric restriction, has been found to have conflicting results on different species [132]. Therefore, it is obvious that systemic rather than targeted therapies can be considered as a more sustainable avenue towards rejuvenation, bringing the spotlight towards the organs that are mostly involved in nutrient regulation.

The “filtration” organ that is mostly associated with metabolism is the liver, as it is responsible for regulating most metabolic pathways including glucose, lipids, and insulin sensitivity. Therefore, it is not surprising to consider the aging liver, particularly liver sinusoidal endothelial cells (LSECs), as contributors to biological aging [133]. Additionally, the liver is linked with the production of methyl compounds, that are involved in DNA methylation, one of the main hallmarks and quantifiers of biological aging [134]. Nevertheless, despite the liver’s central role, only a limited number of studies have been dedicated to the full elucidation of its exact role in aging. Thus, more studies are warranted to understand the effect of the liver on aging and rejuvenation. Nonetheless, it is worth noting that, unlike the kidneys, the liver has unique aging characteristics that mostly manifest over the age of 60 [90], by which time, biological aging hallmarks are already set in most populations, which alleviates its direct implication in aging as a causative agent, or limits it to certain age-associated disease phenotypes. On the other hand, the kidney, the organ that escorts the body in its aging journey, and is responsible for clearing metabolic waste from the bloodstream, can also be implicated in metabolism due to its role in vitamin D synthesis [98], the deficiency of which has been shown to participate in aging, metabolism, and age-associated diseases [98,100].

### 5.2. Senolytic Drugs, the New Pharmacological Focus of Rejuvenation

Although the word senescence is derived from its Latin origin meaning “old”, its implication in propagating the aging process itself has been, up until recently, controversial. This controversy is due to the mostly beneficial views towards senescence, particularly with regards to tissue repair [99], wound healing [95], protection against cancer progression [93], and embryonic development [91]. Therefore, cell senescence was generally regarded as a product of aging, induced by telomere attrition, rather than a possible causative agent. However, a closer examination of the effect of senescent cells on the extracellular milieu, in particular SASP, elucidated the detrimental effects of this phenomenon on aging tissues and organs [101]. The state of proliferation arrest that defines senescent cells is recognized now as a metabolically active state that secrets cytokines, chemokines, proteases, and growth factors that include among others, interleukins (IL-1β, IL-6, IL-8), TGFβ1, and WNT16B, which propagate senescence across tissues and organs [101]. Markers associated with senescence are cyclin-dependent kinase inhibitors p16^INK4a^ and p21CIP1, which are implicated in cell-cycle arrest [91,101]. This clear senescence marker identification, and the advent of animal models lacking p16^INK4a^ in 2001 by two different groups [94,102], or INK-ATTAC transgenic mice that allow for the elimination of cells over-expressing p16^INK4a^ using a targeted drug [96], permitted a clear demonstration of the major effect of cellular senescence on biological aging. Based on these findings, it is clear that cellular senescence is one of the clearest examples of antagonistic pleiotropy [135,136].

A study conducted by Baker et al. (2016) using INK-ATTAC transgenic mice, was able to show that senescent cell ablation extends the lifespan, delays tumorigenesis, and attenuates age-related diseases. This method, however, caused partial and tissue-selective removal of senescent cells, with varying degrees on different organs [96]. In particular, while this method did not affect the colon and the liver, it had a very noticeable effect on the kidneys, the heart, and the adipose tissue. More importantly, senescent cells appear to have specific effects on different organs rather than a uniform systemic role. In this study, it was shown that it caused a hyper-activation of the local renin–angiotensin–aldosterone system (RAAS), progenitor cell dysfunction, and cardiac Sur2a downregulation in kidney, adipose, and heart tissues of old mice, respectively [96]. This variation in SASP mechanistic action opens speculation on the scope of SASP effect and whether this senescence-associated secretome can differ from one tissue to another, and more importantly, if such tissue-specific extracellular vesicles can be traced back in plasma to their original source, thus defining the extent of specific organ SASP on surrounding tissues, organs, and systems.

Several clinical trials are being performed presently to investigate the therapeutic effect of senescent cell ablation on specific organs and medical conditions, such as the kidney (NCT02848131) and osteoarthritis (NCT03513016). Such pharmaceutical interventions include the use of Dasatinib and Quercetin in a controlled manner and over a short period of time. Although the results obtained thus far seem to be promising, given the important role of cell senescence in general physiological functions, such treatment modalities can be used intermittently to remove the excess accumulation of senescent cells that otherwise would overwhelm the immune system. Nevertheless, the effectiveness and long-term benefits of such an approach remains to be determined.

### 5.3. Cellular Reprogramming and Genetic Rejuvenation

When it comes to addressing the concept of “rejuvenation” as opposed to healthy aging, cellular reprogramming is the only one to show a significant reversal of cellular aging hallmarks. Therefore, this promising experimental procedure holds amazing promise for future applications, including system and organ-specific rejuvenation. In 2007, Yamanaka factors were identified after the extensive stripping of transcription factors down to four distinct genes: octamer-binding transcription factor 4 (OCT4), Kruppel-like factor 4 (KLF4), SRY (sex-determining region Y)-box 2 (SOX2), and proto-oncogene MYC (C-MYC), that were dubbed OKSM [137]. Concurrently, Yu et al. introduced another set of four reprogramming factors that included OCT3/4, SOX2, NANOG, and LIN-28 [138]. Since then, other reprogramming agents were presented including micro-RNAs (miRNA) [139,140] and long non-coding RNAs (lncRNA) [141] that aid in the partial reprogramming of somatic cells into different types, otherwise called trans-programming. However, such methods preserve the methylation profile of the original cell, thereby preserving their cellular age profile [139,140]. The importance of cell reprogramming, however, extends beyond aging towards the creation of small organoids that can potentially be harvested and used to treat certain diseases, where preservation of DNA age and methylation are considered beneficial.

Thus far, the major issue that hinders the development of this rejuvenating intervention in a clinical setting is its unpredictability due to the complete reversal of the somatic cell into an induced pluripotent stem cell (iPSC) state. Thus, the manner in which these cells would develop in vivo is highly dependent on extracellular conditions and cannot be guaranteed to adopt a restorative or a regenerative role if surrounded by disease phenotypes. Such inferences can be made based on the numerous studies conducted on stem cell organ and tissue regeneration [141,142,143]. However, the most attractive feature of cell reprogramming is that once differentiated in vitro into specific cell types, cells can retain a youthful methylation and transcriptomic profile, although this might require additional treatment to ensure resetting the methylation signature of the cell [144]. However, successful methods of reverting cellular aging markers, including their methylation signature, without the induction of cell de-differentiation, are yet to be accomplished. Regardless, in vivo partial rejuvenation was effective using short term induction of Yamanaka factors, which restored DNA damage and ameliorated certain aging hallmarks such as deregulated histone and senescence-associated genes leading to a 20% increase in the lifespan of these animal models [145]. This success though does not diminish the possibility of developing other disease conditions or phenotypes, as more comprehensive research is still needed in this area to ensure its safety and translatability into clinical applications.

## 6. Future Outlook and Conclusions

Given the clear differences between a biological system and a mechanical one, any comparison must consider the inherent complexity of biological components, particularly when it comes to the diverse roles that each organ plays and the often-overlapping functions within the system in general. As such, an association of a single organ, such as the kidney, with the complex phenomenon of aging can only be taken as a percent involvement instead of an absolute one. Nevertheless, it is obvious, that contaminant build-up in the working fluid is responsible for significant health issues typically associated with aging. Based on such evidence, specific studies designed to isolate the effect of reduced kidney function on aging are warranted, particularly in the absence of such inferences in the field to date. More importantly, establishing rigorous methods to evaluate the percentage role of specific organs in aging might help establish organ-specific intervention methods that can make use of existing therapeutics and rejuvenation techniques in a more translatable manner.

Organ-specific therapeutics, for example, have seen incredible advances over the past decade, with such methods relying on cell reprogramming techniques to provide long-term solutions for a set of chronic diseases. The use of organoids to restore the functionality of some organs, such as the liver, is one of the most promising therapeutic approaches to date [144,146]; although, there are still many challenges that need to be addressed [147]. Organoid and stem cell-based kidney-specific therapeutics are already advancing presently [148,149]. The main challenge that such treatment modalities face is that they are used as a last resort against severe disease conditions, which entails highly toxic cellular microenvironments. However, repurposing these techniques to treat reduced organ function associated with specific age milestones might eventually be more successful in organ and system maintenance in general.

From a biomedical engineering perspective, future interventions can hold some interesting diagnostic and therapeutic solutions. Such designs are still under investigation, however, the need for an implantable diagnostic tool to successfully measure and report the plasma levels of known toxins, hazardous agents, and disease markers, can potentially help prevent and resolve disease conditions at their early stages [150]. Particularly since, once the symptoms of aging associated diseases manifest, existing therapeutics become increasingly limited. This is particularly important in the context of rejuvenation studies, as aging tends to take hold before any specific disease phenotype is manifested. Further advancements in implantable technology can potentially be actively involved, instead of passively reporting diagnostic data wirelessly [151]. Such devices can potentially remove toxic components in a selective manner and store them until it reaches saturation, only to be removed and replaced with another membrane. This additional filtration mechanism could possibly prevent an extensive list of chronic diseases while providing medical screening and diagnostic tools. Presently though, advances in medical research are far more advanced than their counterparts in biomedical engineering, which is fraught with several challenges and restrictions that hinders their swift development [152]. In particular, implantable devices have to be biocompatible while ensuring an optimum interface with their surrounding tissue and fluids [153]. Tissue build-up is a common problem that such devices face, which causes a reduction in the efficacy of sensors and detectors in an exponential manner [154]. Additionally, other engineering concerns include battery lifecycle [155], degradation over time [151], and information transmissibility [156]. While some of these issues have been addressed by the advent of several biocompatible and non-toxic implantable materials, the design of a completely independent long-term implantable device that relies on implantable in vivo energy-harvesting fuel cells is still challenging. Thus, while future medical solutions can be very promising, present biomedical knowledge can successfully tackle aging diseases successfully once efforts are directed towards biological systems maintenance instead of damage control.

In conclusion, we herein attempt to provide a general platform to address aging, where we stress the importance of having standardized biometrics used in rejuvenation studies. Such a platform can potentially allow for the assessment and comparison of any advances made in the field and build upon their success. More importantly, we herein attempt to shine a light on a rejuvenation avenue that has been largely overlooked. The new methodology targets the kidney specifically, which has an aging profile that coincides with overall systemic aging, based on the possible role of filtration organs and contaminant build up on aging. This hypothesis is further examined by reviewing the involvement of the kidney in various known aging factors and after listing existing rejuvenation methods, the possibility of focusing some of these on kidney maintenance becomes very interesting within the context of systemic rejuvenation. The possible involvement of the kidney in aging brings about the need for a large body of research to confirm its direct role in this process. Additionally, once established, numerous rejuvenation methods can be explored in order to maintain the functionality of the kidney as a filtration system or bring about diagnostic or other engineering solutions to aid with the filtration process. However, given that this particular topic has not been explored so far, and in the absence of any direct findings on the possible involvement of the kidney in aging, this presents a major challenge or a limitation in the present review, as this hypothesis hinges on the amazing results presented by Mehdipour et al., (2020), where replacement of plasma with a mixture of saline and albumin was sufficient to produce the same rejuvenation effects observed in heterochronic parabiosis. Additionally, the role of the central nervous system, and in particular the effect of hypothalamus aging on energy homeostasis, hormone balance, circadian rhythm, nutrient sensing dysfunction, stem cell exhaustion, loss of proteostasis, and epigenetic alterations was not discussed [157,158], focusing more on major pathways of rejuvenation studies instead. Thus, it is obvious that several studies are necessary to elucidate the exact role of filtration on aging, but it is our belief that such an approach is indeed warranted and merits further exploration.

## Figures and Tables

**Table 1:** Clusters of protein trajectories that reflect changes with age coinciding with the onset of aging, and the top 10 plasma proteins involved in them [72].

Top 10 Plasma Protein Pathways Increasing with Age			Top 10 Plasma Proteins Pathways Decreasing with Age	
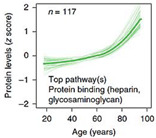	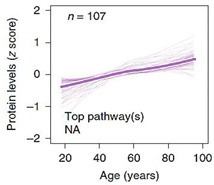	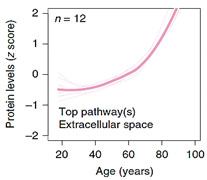	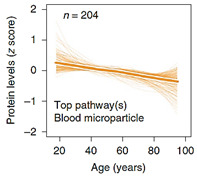	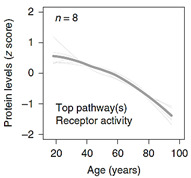
**Extracellular Region**	**Chondroitin Sulfate Biosynthesis**	**Extracellular Space**	**Extracellular Space**	**Transmembrane Receptor Activity**
ADAMTS5/ASPN/C1QTNF1/…/ TREML4/TTN/VEGFA/VIT	CHST9/DCN/BGN/CHST11	CCDC80/FSTL3/GDF15/MMP12/NPPB/PTN/SVEP1/WFDC2	ACE2/ADAMTS3/AHSG/AIMP1/…/SPINT1/TF/TNXB/UBB	EGFR/KDR/RET
**Extracellular Region Part**	**Heparan Sulfate Metabolism**	**Fibronectin Binding**	**Blood Microparticle**	**Tyrosine Kinase Activity**
ADAMTS5/ASPN/…/TNFSF15/TTN/VEGFA/VIT	DCN/BGN/GPC6/GLCE/HS3ST3A1	CCDC80/FSTL3	AHSG/APOL1/CFB/…/IGLL1/ITIH1/PLG/SERPINC1/TF	EGFR/KDR
**Extracellular Space**	**Chondroitin Sulfate Metabolism**	**Extracellular Region**	**Fibrillar Collagen Trimer**	**Integrin Binding**
ADAMTS5/C1QTNF1/…/TNFRSF11B/TNFSF15/VEGFA	CHST9/DCN/BGN/GPC6/CHST11	CCDC80/CHRDL1/FSTL3/…/PTN/RNASE1/SVEP1/WFDC2	COL11A2/COL1A1/TNXB	ADAMTS13/EGFR/KDR
**Proteinaceous Extracellular Matrix**	**Regulation of IFNG Signaling**	**Extracellular Region Part**	**Banded Collagen Fibril**	**Protein Kinase Activity**
ADAMTS5/ASPN/CHI3L1/…/TNFRSF11B/VEGFA/VIT	STAT1/SOCS3/PTPN11	CCDC80/…/MMP12/NPPB/PTN/RNASE1/SVEP1/WFDC2	COL11A2/COL1A1/TNXB	EGFR/KDR/RET
**Glycosaminoglycan binding**	**Glycosaminoglycan Metabolism**	**Basement Membrane**	**Extracellular Region**	**Midgut Development**
ADAMTS5/CXCL10/GREM2/…/SFRP1/THBS3/VEGFA/VIT	CHST9/DCN/BGN/GPC6/CHST11/GLCE/HS3ST3A1	CCDC80/PTN	ACE2/ADAMTS3/AGER/…/TMEM132A/TNR/TNXB/UBB	EGFR/RET
**Heparin Binding**	**Interleukin-6 signaling**	**Extracellular Matrix Component**	**Extracellular Region Part**	**Hormone Binding**
ADAMTS5/CXCL10/GREM2/…/SFRP1/THBS3/VEGFA	STAT1/SOCS3/PTPN11	CCDC80/PTN	ACE2/ADAMTS3/…/TF/TMEM132A/TNR/TNXB/UBB	EGFR/GHR
**Sulfur Compound Binding**	**Defective B4GALT7, Progeroid**	**Proteinaceous Extracellular Space**	**Protein Activation Cascade**	**Protein Complex Binding**
ADAMTS5/CD34/…/SFRP1/THBS3/VEGFA	DCN/BGN/GPC6	CCDC80/MMP12/PTN	C1RL/CFB/CFP/…/IGLL1/MASP1/MBL2/SERPINC1	ADAMTS13/CTSV/EGFR/KDR
**G-protein Coupled Receptor Binding**	**Defective B3GAT3**	**BMP Signaling Pathway**	**Complex of Collagen Trimers**	**Transmembrane Signaling Receptor Activity**
CCL3/CXCL10/CXCL16/…/NPW/POMC/PPY/RSPO3/SFRP1	DCN/BGN/GPC6	CHRDL1/FSTL3/GDF15	COL11A2/COL1A1/TNXB	EGFR/GHR/KDR/RET
**Chitin Binding**	**Defective B3GALT6**	**Response to BMP**	**Extracellular Exosome**	**Macromolecular Complex Binding**
CHI3L1/CHIT1/CTBS	DCN/BGN/GPC6	CHRDL1/FSTL3/GDF15	ACE2/ADAMTS3/…//ST3GAL6/TF/TMEM132A/TNXB/UBB	EGFR/GHR/KDR/RET
**Extracellular Matrix**	**Particulate Exogenous Antigens**	**Cellular Response to BMP**	**Extracellular Organelle**	**Protein Tyrosine Kinase Activity**
ADAMTS5/ASPN/…/TIMP4/TNFRSF11B/VEGFA/VIT	CD36/ITGAV	CHRDL1/FSTL3/GDF15	ACE2/ADAMTS3/AHSG/…/TF/TMEM132A/TNXB/UBB	ADAMTS13/CTSV/EGFR/KDR

**Table 2:** The molecular weight of plasma proteins that are affected with age.

Plasma Proteins that Increase with Age	Abbreviation	Molecular Weight (kda)	Ref.	Plasma Proteins That Decrease with Age	Abbreviation	Molecular Weight (kda)	Ref.
**Motilin**	MLN	5.19	[90]	**Cadherin-13**	CDH13	7.57	[91]
**Eotaxin**	CCL11	10.73	[92]	**Apelin**	APLN	8.57	[93]
**C-C** **Motif Chemokine 19**	CCL19	10.99	[92]	**Osteocalcin**	OCN(BGLAP)	10.96	[94]
**C-C** **Motif Chemokine 2**	CCL2	11.03	[92]	**Somatoliberin**	GHRH	12.45	[95]
**β-2-** **Microglobulin**	B2M	11.73	[96]	**Oxytocin-Neurophysin 1**	OXT	12.72	[97]
**Natriuretic Peptides B**	NPPB	14.73	[98]	**Granulocyte-Macrophage Colony-Stimulating Factor**	CSF2	16.3	[99]
**Pleiotrophin**	PTN	18.95	[90,98]	**Metalloproteinase Inhibitor 2**	TIMP2	24.4	[99]
**A Disintegrin And Metalloproteinase with Thrombospondin Motifs 5**	ADAMTS5	21.7	[98]	**Growth Differentiation Factor 11**	GDF11	45.1	[100]
**Sclerostin**	SOST	24.03	[90,98]	**Extracellular Nicotinamide Phosphoribosyltransferase**	eNAMPT	55.53	[101]
**Growth Differentiation Factor 15**	GDF15	34.15	[90,98]	**Thrombospondin-4**	THBS4	96.03	[102]
**Cathepsin L2**	CTSV	37.33	[98]	**Proto-Oncogene Tyrosine-Protein Kinase Receptor Ret**	RET	124.39	[90]
**Adp Ribosylation Factor Interacting Protein 2**	ARFIP2	37.86	[90]	**Immunoglobulin Superfamily Dcc Subclass Member 4**	IGDCC4	134.23	[90]
**Haptoglobin**	HP	45.21	[92]				
**Chordin-Like Protein 1**	CHRDL1	51.18	[98]				
**Macrophage Metalloelastase**	MMP12	54.01	[98]				
**Egf-Containing Fibulin-Like Extracellular Matrix Protein 1**	EFEMP1	54.65	[98]				
**Scavenger Receptor Class F Member 2**	SCARF2	92.4	[90]				

## Data Availability

Not applicable.
